# Editorial: Growth hormone in the nervous system: from biology to therapy

**DOI:** 10.3389/fnins.2023.1297505

**Published:** 2023-10-16

**Authors:** Enrique Juarez Aguilar

**Affiliations:** Instituto de Ciencias de la Salud, Universidad Veracruzana, Xalapa, Veracruz, Mexico

**Keywords:** growth hormone, nervous system, biology, therapy, function

The role of growth hormone (GH) in development and function of the nervous system continues to be a relevant area of research. Despite a large amount of scientific literature on the subject, gaps in our knowledge still need to be filled before this hormone can be therapeutically used for the treatment of injuries and diseases of the nervous system.

Much evidence of the possible role of GH in the nervous system has been generated from animal models of GH deficiency. The absence of this molecule alters neural populations during nervous system development, especially in the brain, causing behavioral changes during both childhood and adulthood. However, effects of GH deficiency on brain anatomy and function during childhood are still unclear. In the present Research Topic, Zhou et al. evaluated such changes in pediatric patients with GH deficiency using nuclear magnetic resonance imaging, presenting evidence of the relevant role of this hormone in brain anatomy and function during childhood.

The therapeutic use of GH for the treatment of pediatric GH deficiency is widely accepted, but the use of new long-acting formulations of recombinant GH requires verification of its safety and efficacy. Interestingly, Wu et al. evaluated the effect of chronic treatment with a polyethylene glycol-modified GH (PEG-GH) preparation in pediatric patients with GH deficiency by measuring the degree of epithelial cell vacuolization in the choroid plexus in the brain in cynomolgus monkeys and analyzing safety data from pediatric patients with GH deficiency. Although the work of Wu et al. is not directly related to brain function, their data on the safety of treatment with PEG-GH in the choroid plexus suggest that this preparation may safely counteract negative effects on brain anatomy and function in pediatric patients with GH deficiency (Zhou et al.).

Pediatric GH deficiency can be idiopathic and transient. This condition can occur from trauma and cerebral vascular events, generating alterations of cognition, motor function, and even mood. Various studies demonstrated the effectiveness of treatment with GH to counteract these deleterious effects on the brain. However, little is known about the role of GH in lesions of the peripheral nervous system. In this Research Topic, Martínez-Moreno et al. evaluated the impact of GH, alone and in combination with gonadotropin-releasing hormone, on damage that was caused by injury to the thoracic spine. Their results suggest a relevant role for GH both alone and combined with gonadotropin-releasing hormone in regulating the repair response in the peripheral nervous system similarly to the brain.

Low levels of GH during aging affect cognition, and GH administration restores it, further underscoring the important role of GH in central nervous system function. In their review, Blackmore and Waters discuss the role of exercise in regulating GH levels to counteract adverse effects of GH deficiency in the brain during aging and its role in recovering the aged brain after damage.

These studies and the scholarly review in this Research Topic represent examples of research that is currently being conducted to more thoroughly understand the role of GH in the nervous system and the ways in which its therapeutic use can favor the maintenance and restoration of nervous system function.

## Author contributions

EJA: Writing—original draft.

